# The Role of Microsatellite Instability in Endometrial Hyperplasia and Risk of Carcinoma Development

**DOI:** 10.3390/biomedicines13122953

**Published:** 2025-11-30

**Authors:** Angelina Mollova-Kyosebekirova, Ekaterina Uchikova, Anna Mihaylova, Mariya Koleva-Ivanova, Mariana Parahuleva, Nikoleta Parahuleva

**Affiliations:** 1 Department of General and Clinical Pathology, Medical University of Plovdiv, 4002 Plovdiv, Bulgaria; angelina.mollova@mu-plovdiv.bg (A.M.-K.); mariya.koleva@mu-plovdiv.bg (M.K.-I.); 2 Department of Obstetrics and Gynecology, Faculty of Medicine, Medical University of Plovdiv, 4000 Plovdiv, Bulgaria; ekaterina.uchikova@mu-plovdiv.bg (E.U.); nikoleta.parahuleva@mu-plovdiv.bg (N.P.); 3 Department of Healthcare Management, Faculty of Public Health, Medical University of Plovdiv, 4000 Plovdiv, Bulgaria; 4 Universitätsklinikum Gießen und Marburg Standort Marburg, 35043 Marburg, Germany; mariana.parahuleva@prof-parahuleva.de

**Keywords:** endometrial hyperplasia, microsatellite instability, mismatch repair deficiency, MLH1, PMS2, MSH6, Lynch syndrome, endometrial carcinoma, immunohistochemistry, epigenetic instability

## Abstract

Background: Endometrial hyperplasia (EH) represents a precursor lesion in the development of endometrial carcinoma, particularly the endometrioid subtype. Among the molecular pathways involved, microsatellite instability (MSI) resulting from DNA mismatch repair (MMR) deficiency has gained increasing attention as an early event in endometrial carcinogenesis. Objective: This study aimed to evaluate the expression of key MMR proteins (MLH1, PMS2, MSH2, and MSH6) in endometrial hyperplasia without atypia and endometrial atypical hyperplasia/endometrioid intraepithelial neoplasia (EAH/EIN) to determine the prevalence and potential implications of MMR deficiency at the precancerous stage. Methods: Fifty-six cases of EH were analyzed, including 28 endometrial hyperplasia without atypia and 28 EAH/EIN. Immunohistochemical (IHC) analysis was performed to assess the nuclear expression of MMR proteins. Loss of expression was defined as complete absence of epithelial nuclear staining with retained stromal positivity. Results: MMR protein expression was retained in all cases of endometrial hyperplasia without atypia, while total loss of one or more MMR proteins was observed in 3 of 28 (10.7%) EAH/EIN. The most frequent pattern involved concurrent MLH1/PMS2 loss, consistent with sporadic MLH1 promoter hypermethylation. One case exhibited isolated MSH6 loss, suggesting a potential Lynch syndrome, and another showed combined MSH6/PMS2 loss. Conclusions: MMR deficiency appears confined to atypical EH, supporting its role as an early molecular alteration in the neoplastic sequence leading to endometrioid carcinoma. Identification of abnormal MMR expression in EH may facilitate risk stratification, guide reflex testing for MLH1 methylation, and prompt genetic counseling for hereditary cancer predisposition.

## 1. Introduction

EH represents a spectrum of histopathological changes within the endometrium, characterized by proliferation of endometrial glands of irregular size and shape, often accompanied by an increased gland/stroma ratio [1]. It arises because of prolonged, unopposed estrogenic stimulation and constitutes an important precursor lesion in the development of endometrial carcinoma, particularly the endometrioid type [2,3]. The classification and grading of endometrial hyperplasia have undergone significant refinement, most recently in the 2014 World Health Organization (WHO) system, which emphasizes the distinction between hyperplasia without atypia and EAH/EIN to better predict malignant potential [4,5]. Understanding its etiology, risk factors, and histopathological subtypes is crucial for accurate diagnosis, prognostication, and the development of appropriate management strategies [6].

Microsatellite instability (MSI), a molecular hallmark of defective DNA mismatch repair (MMR), arises most often through alterations in the MMR proteins MLH1, MSH2, MSH6, and PMS2 [7]. While extensively described in endometrial carcinoma—particularly within the endometrioid subtype, where it is observed in approximately one quarter of cases [8]—its occurrence has also been documented in EH, though less frequently.

Evidence indicates that MSI is more often detected in EAH/EIN, compared to hyperplasia without atypia. This suggests a potential role of MSI in the neoplastic progression pathway from precursor lesion to carcinoma [9,10]. In many cases, MSI within hyperplastic endometrium reflects MLH1 promoter hypermethylation, paralleling the mechanisms observed in sporadic endometrial carcinomas [11].

From a prognostic standpoint, the presence of MSI in EH is regarded as a molecular marker that may signify an increased likelihood of malignant transformation. However, its independent predictive value remains controversial, since some studies propose that MSI positive hyperplasia does not necessarily demonstrate a more aggressive clinical course, but rather shares early molecular changes with carcinoma [12]. Increasingly, the recognition of MSI status is of clinical interest, as endometrial carcinomas with high MSI show enhanced responsiveness to immune checkpoint inhibitors. This raises the possibility that MSI testing in advanced or EAH/EIN may contribute to more refined risk stratification and future therapeutic considerations [13].

In addition, identification of MMR deficiency in EH may have implications for Lynch syndrome (hereditary nonpolyposis colorectal cancer, HNPCC). Isolated loss of MSH6 or PMS2, and particularly unusual staining patterns, should raise the suspicion of a germline defect rather than sporadic MLH1 promoter hypermethylation. Given that endometrial carcinoma is often the sentinel malignancy in women with Lynch syndrome, recognition of MMR protein loss at the hyperplasia stage could prompt timely genetic counseling and surveillance strategies, not only for the patient but also for at risk relatives [14].

Recent molecular and translational studies across diverse biological systems emphasize the universal importance of genomic and epigenetic stability in maintaining cellular homeostasis and preventing malignant transformation. Investigations in plant models, such as *Crithmum maritimum* and *Echinophora* species, have demonstrated how environmental and oxidative stress can induce DNA methylation alterations affecting repetitive and regulatory regions [15,16]. Comparable epigenetic mechanisms are implicated in endometrial carcinogenesis, where MLH1 promoter hypermethylation disrupts mismatch repair fidelity and promotes microsatellite instability. Moreover, experimental data from receptormediated and antioxidant assays using *Asplenium* and *Marrubium* extracts support the link between oxidative stress and genomic instability [17,18]. These crossdisciplinary insights reinforce the concept that microsatellite instability in endometrial hyperplasia represents not only a localized molecular event but part of a broader biological paradigm of stress-induced genomic instability leading to carcinoma development.

Building on these insights, our study aimed to evaluate the occurrence of mismatch repair deficiency within EH as an early molecular event predisposing to carcinoma. By analyzing the expression patterns of key MMR proteins—MLH1, PMS2, MSH2, and MSH6—we sought to distinguish between sporadic and potentially hereditary cases and to explore the clinical implications of MMR loss at the precancerous stage. This molecular characterization provides a framework for understanding how microsatellite instability contributes to endometrial tumorigenesis and supports the implementation of targeted diagnostic strategies.

Several studies have shown an absence of MMR deficiency in endometrial hyperplasia without atypia, whereas a small but significant proportion of EAH/EIN lesions demonstrate loss of MMR proteins.

Vierkoetter and his team examined 112 EIN lesions and found MMR loss in 4.5% of cases, predominantly involving MLH1/PMS2 co-loss associated with MLH1 promoter hypermethylation, indicating a sporadic mechanism rather than a hereditary one. MLH1 hypermethylation was confirmed in all MLH1-absent cases, supporting the conclusion that sporadic epigenetic silencing is the dominant pathway in precursor lesions [19].

Similarly, Watkins et al. analyzed 118 unselected EIN/AH biopsies and reported MMR deficiency in approximately 3% of cases, again primarily due to MLH1/PMS2 co-loss linked to promoter hypermethylation. In that study, concordance between precursor lesion and associated carcinoma was high, particularly in Lynch-associated lesions where loss patterns were stable across progression [14]. Together, these findings reinforce that MMR deficiency is uncommon but detectable in EIN/EAH, and seldom present in non-atypical hyperplasia.

Recent work has emphasized that EIN/EAH frequently represents a neoplastic precursor with molecular mutations paralleling those found in endometrioid carcinoma. Leino A. et al., in a molecular profiling study of 40 EIN/EAH lesions, identified MMR deficiency in 5 cases, as well as rare POLE-mutated and p53-abnormal lesions. Most cases (over 80%) belonged to the “no specific molecular profile” (NSMP) category, consistent with patterns seen in low-grade endometrioid carcinoma. Importantly, all MMR-deficient or p53-abnormal EIN lesions demonstrated persistent disease or progression, underscoring the prognostic relevance of molecular subtyping [20].

## 2. Materials and Methods

### 2.1. Study Design and Case Selection

A total of 56 cases of EH were retrospectively analyzed from the Bulgarian population. The cohort included 28 cases of endometrial hyperplasia without atypia and 28 EAH/EIN, diagnosed between 2023–2025. All samples were obtained from patients who provided written informed consent for the use of tissue material for research purposes. The study was conducted in accordance with institutional ethical standards and approved by the Local Ethics Committee of the Medical University of Plovdiv, Bulgaria (PKHE3/28 October 2024), following the principles outlined in the Declaration of Helsinki.

### 2.2. Histopathological Evaluation

**Figure 1:**
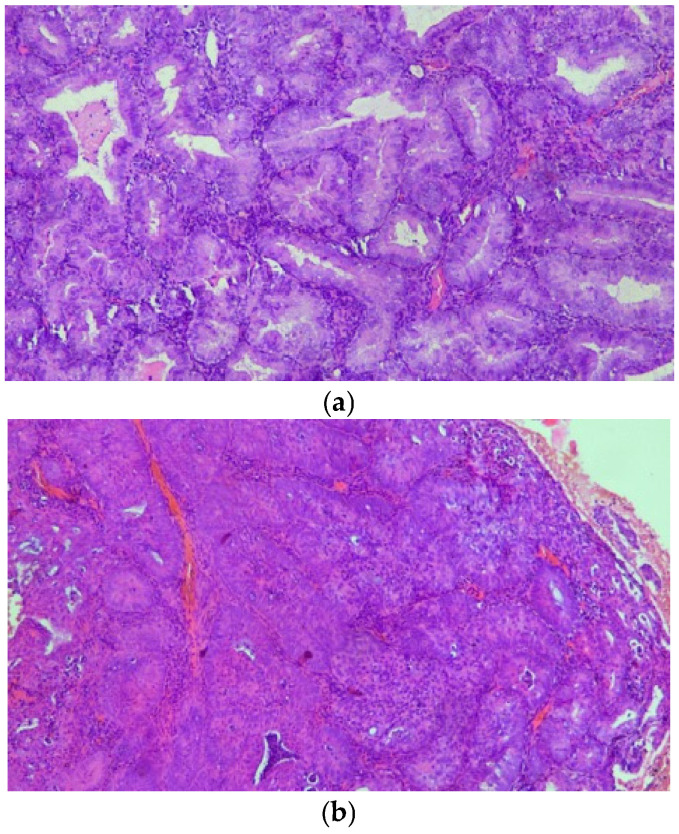
(**a**,**b**) On H&E stain, AH/EIN shows densely packed glands with irregular or complex architectural contours, lined by epithelium demonstrating cytologic atypia.

All cases were reviewed on hematoxylin and eosin (H&E)-stained slides. Diagnoses were confirmed independently by two experienced pathologists, and lesions were classified according to the 2014 World Health Organization (WHO) criteria. (Figure 1) The classification distinguished between hyperplasia without atypia and EAH/EIN.

### 2.3. Immunohistochemistry

Formalin fixed, paraffin embedded (FFPE) tissue blocks were used for immunohistochemical (IHC) analysis of DNA mismatch repair (MMR) proteins. Serial 4 μm sections were stained using standard automated protocols for MLH1, PMS2, MSH2, and MSH6 (antibody clones and sources may be specified if required by the journal). Nuclear staining in glandular epithelial cells was evaluated. Retained expression was defined as intact nuclear staining in both epithelial and stromal cells. Complete absence of epithelial nuclear staining, with preserved stromal nuclear staining serving as an internal positive control, was interpreted as loss of expression.

### 2.4. Data Interpretation

Cases showing concurrent loss of MLH1 and PMS2 were considered indicative of sporadic MLH1 promoter hypermethylation. Isolated loss of MSH6 or PMS2 was interpreted as suggestive of a possible germline mutation consistent with Lynch syndrome. Ambiguous or mixed staining patterns were reevaluated to exclude technical artifacts.

## 3. Results

### 3.1. MMR Protein Expression in Endometrial Hyperplasia Without Atypia and EAH/EIN

Immunohistochemical evaluation of the four DNA, MMR proteins—MLH1, PMS2, MSH2, and MSH6—was successfully performed in all 56 cases of EH. Among the 28 cases of endometrial hyperplasia without atypia, nuclear expression of all MMR proteins was retained, indicating the absence of mismatch repair deficiency (0%).

In contrast, loss of MMR protein expression was identified in 3 out of 28 EAH/EIN cases (10.7%). The remaining 25 EAH/EIN cases demonstrated intact nuclear staining for all proteins. These findings indicate that MMR deficiency occurs exclusively within the EAH/EIN subgroup and is absent in endometrial hyperplasia without atypia.

### 3.2. Patterns of MMR Protein Loss

Among the three MMR deficient EAH/EIN cases, the most frequent pattern was concurrent loss of MLH1 and PMS2, observed in two cases. This profile is consistent with sporadic MLH1 promoter hypermethylation, representing the predominant molecular mechanism of MMR deficiency in endometrial neoplasia.

**Figure 2:**
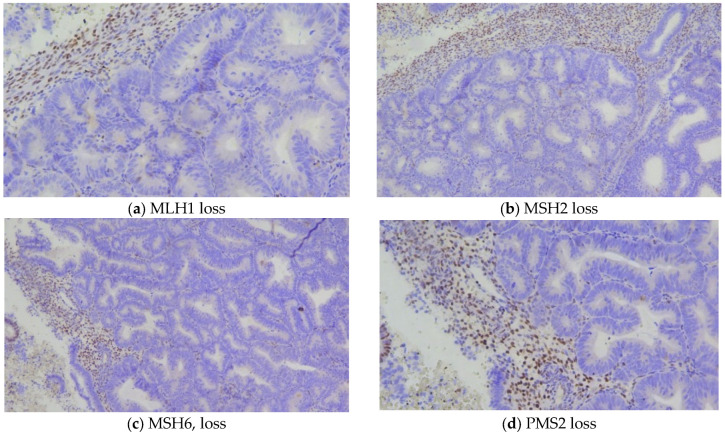
General view of areas with EAH/EIN, showing loss of MSI expression, with positive internal stromal control.

One case demonstrated isolated loss of MSH6, a pattern suggestive of a possible germline mutation associated with Lynch syndrome. Another case exhibited a combined loss of MSH6 and PMS2, an uncommon finding warranting further verification to exclude technical artifact or multiple pathway alterations (Figure 2).

### 3.3. Clinical and Diagnostic Implications

The identification of MMR deficiency exclusively within EAH/EIN supports the hypothesis that MMR loss may represent an early molecular event in the neoplastic progression toward endometrioid endometrial carcinoma. Recognition of MLH1/PMS2 co-loss should prompt reflex testing for MLH1 promoter methylation to differentiate sporadic from hereditary cases. Similarly, isolated MSH6 or PMS2 loss should trigger consideration of genetic counseling and germline testing to identify individuals at risk for Lynch syndrome.

## 4. Discussion

### 4.1. Biological Significance of MMR Deficiency in EH

In this study, we evaluated the expression of the four key MMR proteins—MLH1, PMS2, MSH2, and MSH6—in typical and EAH/EIN. Our findings revealed that MMR deficiency was absent in all cases of endometrial hyperplasia without atypia but present in 10.7% of EAH/EIN, indicating a biological link between MMR loss and precancerous transformation. This pattern supports the concept that defects in DNA repair mechanisms occur early in the carcinogenic sequence, contributing to genomic instability and progression toward endometrioid endometrial carcinoma.

The observed loss of MLH1/PMS2 expression aligns with previous evidence showing that sporadic MLH1 promoter hypermethylation represents the most frequent cause of MMR deficiency in endometrial neoplasia. This mechanism disrupts DNA repair fidelity, leading to microsatellite instability (MSI) and accumulation of somatic mutations that drive neoplastic transformation.

### 4.2. Implications for Lynch Syndrome Detection

Of note, one EAH/EIN case exhibited isolated MSH6 loss, a hallmark pattern associated with Lynch syndrome. Because endometrial carcinoma is often the sentinel malignancy in women with Lynch syndrome, the identification of such staining patterns at the hyperplasia stage may provide an opportunity for early detection and genetic counseling. Similarly, the unusual case showing combined MSH6 and PMS2 loss warrants further analysis, as mixed or atypical immunohistochemical patterns may indicate technical artifact or the coexistence of multiple molecular alterations. Repeat IHC testing and, where feasible, molecular validation should be considered to confirm the result.

### 4.3. Clinical and Prognostic Relevance

From a clinical perspective, the presence of MMR deficiency in EAH/EIN reinforces its role as a potential biomarker for malignant transformation. While some studies have reported that MSI positive hyperplasia does not necessarily predict aggressive behavior, its detection underscores shared molecular features between EH and endometrioid carcinoma. Importantly, endometrial cancers with high MSI (MSIH) status have demonstrated enhanced responsiveness to immune checkpoint inhibitors, highlighting the growing therapeutic relevance of MSI and MMR testing. Therefore, assessing MMR status even in precancerous lesions may support personalized management and future integration into targeted treatment strategies.

### 4.4. Diagnostic Recommendations

Our findings support the inclusion of MMR immunohistochemistry in the routine diagnostic evaluation of EAH/EIN. The identification of MLH1/PMS2 co-loss should prompt reflex MLH1 promoter methylation testing to distinguish sporadic from hereditary cases. Detection of isolated MSH6 or PMS2 loss should trigger referral for genetic counseling and possible germline testing. Incorporating MMR IHC screening at the hyperplasia stage offers valuable information for risk stratification, early identification of Lynch syndrome, and potential therapeutic planning.

### 4.5. Study Limitations and Future Directions

The main limitation of this study is the relatively small cohort size, which reflects the rarity of confirmed EAH/EIN cases with MMR loss. Larger multicentric studies are warranted to validate these findings and establish standardized algorithms for the molecular assessment of EH. Future research integrating methylation analysis, MSI PCR, and next generation sequencing may further clarify the temporal sequence of MMR loss during endometrial carcinogenesis.

### 4.6. Speculative Insights into Carcinogenesis in EH

While our findings can support MMR deficiency as an early molecular event in the progression from EAH/EIN to endometrial carcinoma, it is likely that MMR loss represents only one of several potential oncogenic pathways. The predominance of MLH1/PMS2 loss in sporadic cases suggests a key role for epigenetic silencing in initiating genomic instability; however, the absence of MMR deficiency in the majority of atypical hyperplasia cases implies that alternative driver mutations may contribute to neoplastic transformation.

Potential mechanisms may include activating mutations in PIK3CA, KRAS, or PTEN, as previously reported in endometrial precancerous lesions. These mutations could cooperate with or act independently of DNA repair defects to promote cellular proliferation, evasion of apoptosis, and progression to carcinoma. Moreover, the observed isolated loss of MSH6 or combined MSH6/PMS2 deficiency raises the possibility of heterogeneous molecular pathways, where germline mutations, somatic epigenetic alterations, or multiple concurrent alterations interact to drive tumorigenesis.

## 5. Conclusions

MMR deficiency was absent in endometrial hyperplasia without atypia but detected in EAH/EIN cases, due to MLH1/PMS2 co-loss consistent with sporadic MLH1 promoter hypermethylation. Occasional cases with isolated or unusual loss patterns raise the possibility of Lynch syndrome and highlight the need for careful interpretation and appropriate reflex testing. These findings suggest that assessment of MMR status in atypical endometrial hyperplasia may provide valuable information for risk stratification, early identification of hereditary cancer predisposition, and potential therapeutic considerations in the context of emerging immunotherapies.

## Data Availability

The original contributions presented in this study are included in the article. Further inquiries can be directed to the corresponding author.
